# Supplementary material for: Asymmetric eROSITA bubbles as the evidence of a circumgalactic medium wind
Source: Nat Commun. 2023 Feb 11;14:781. doi: 10.1038/s41467-023-36478-0 (PMC9922264; doi:10.1038/s41467-023-36478-0)
Supplement: Supplementary file 1 — Supplementary Information [file 41467_2023_36478_MOESM1_ESM.pdf]

# Supplementary Information for "Asymmetric eROSITA bubbles as the evidence of a circumgalactic medium wind"

**Guobin Mou[1,2*], Dongze Sun[3], Taotao Fang[4*], Wei Wang[1,2*], Ruiyu Zhang[5], Feng Yuan[6], Yoshiaki Sofue[7], Tinggui Wang[8], and Zhicheng He[8]**

[1]School of Physics and Technology, Wuhan University, Wuhan 430072, China
[2]WHU-NAOC Joint Center for Astronomy, Wuhan University, Wuhan 430072, China
[3]California Institute of Technology, Pasadena, CA 91125, USA
[4]Department of Astronomy, Xiamen University, Xiamen, Fujian 361005, China
[5]School of Physics, Henan Normal University, Xinxiang 453007, China
[6]Shanghai Astronomical Observatory, Chinese Academy of Sciences, 80 Nandan Road, Shanghai 200030, China
[7]Institute of Astronomy, The University of Tokyo, Mitaka, Tokyo 181-0015, Japan
[8]School of Astronomy and Space Science, University of Science and Technology of China, Hefei 230026, China
[*]Corresponding authors: gbmou@whu.edu.cn; fangt@xmu.edu.cn; wangwei2017@whu.edu.cn

**Supplementary Note 1: Coordinates of the Northern eROSITA bubble's Edge**

Coordinates $(l, b)$ of the points marking the contours of the Northern eROSITA bubble (shown in Figure 2a) are listed in Table 1:

**Supplementary Note 2: Small-scale Simulations**

The small-scale 3D simulations are aimed at capturing the process of CMZ regulating the nuclear outflow, and generating the parameters of nuclear outflow injected into the Galactic scale simulations (see Supplementary Figure 2). The biconical outflow is supersonic with a ram pressure much higher than that of ISM/CGM, transportation of material and energy given by small-scale simulations is one-way (outward), and the large-scale environment outside does not affect the small-scale domain.

**Supplementary Note 3: Dependence of the Results on the Kinetic Luminosity $L_k$**

The results for different $L_k$ in the CGM wind model are shown in Supplementary Figure 3. The surface brightness of simulated eRBs is significantly affected by $L_k$. Given the halo temperature of $2 \times 10^6$ K[1], the post-shock CGM (or CGM wind) density and temperature required for modeling the eRBs[2,3], there is no much space for adjusting the kinetic luminosity. Although it can take a longer time for a lower $L_k$ to supply enough energy to inflate bubbles, the temperature of post-shock CGM (especially the southern halo which does not suffer much from the CGM wind) will become lower, leading to dimmer eRBs and deviating from the X-ray spectral shape, and vice versa. Based on our preliminary tests, the approximate range of the averaged kinetic luminosity should be between $2 \times 10^{41}$ and $8 \times 10^{41}$ erg s$^{-1}$. One could adopt a lower

**Table 1.** Coordinates $(l, b)$ of the Northern eROSITA bubble's Edge.

| | | | | | | | | | | |
|---|---|---|---|---|---|---|---|---|---|---|
| $l$ | 29.3 | 36.1 | 37.5 | 39.0 | 36.4 | 6.1 | -56.1 | -66.8 | -52.0 | -53.2 |
| $b$ | 8.2 | 17.2 | 34.6 | 55.8 | 66.5 | 78.3 | 76.2 | 69.5 | 48.6 | 61.2 |
| $l$ | -41.5 | 0.0 | -1.6 | 9.0 | 11.7 | 17.0 | -45.0 | -60.0 | -53.0 | |
| $b$ | 66.8 | 60.0 | 48.7 | 35.2 | 25.5 | 15.2 | 30.0 | 30.0 | 20.0 | |

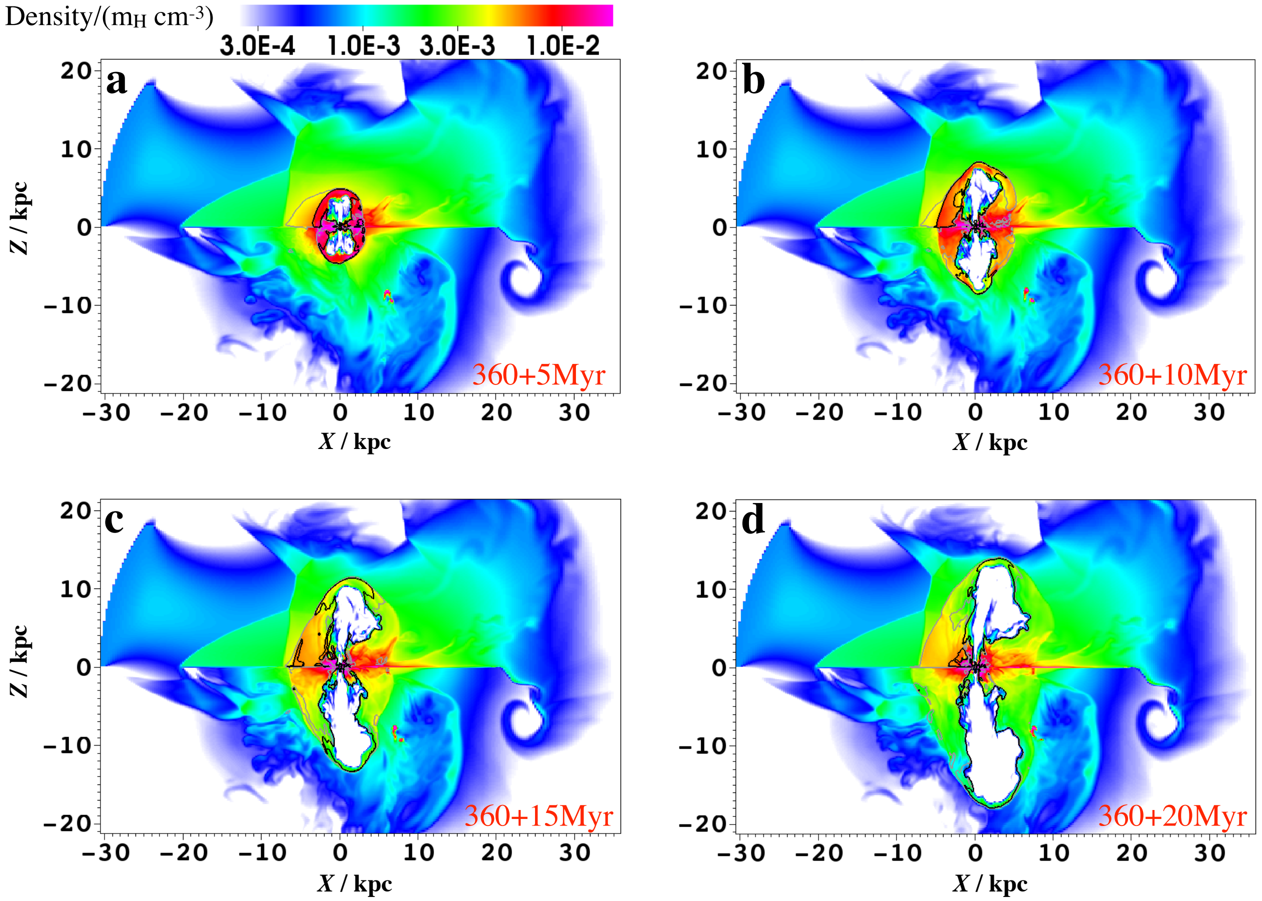


**Supplementary Figure 1. Snapshots of density evolution in the fiducial run of the CGM wind model.** Coordinate values are in units of kpc. The grey and black lines denote isotherms of $3 \times 10^6$ and $4 \times 10^6$ K, respectively.

kinetic luminosity by setting a higher initial CGM temperature (e.g., $2.5 \times 10^6$ K[4]). However, this will result in a significantly low brightness contrast of the eRBs in background compared with observations.

**Supplementary Note 4: Dependence of the Results on the Velocities of the CGM Wind**

We tested the results with different injection velocities of the CGM wind $v_{\rm CGM}$, and ploted the representative cases of the weak and strong CGM wind in Supplementary Figure 4.

**Supplementary Note 5: Effect of CGM Temperature**

For the non-axisymmetric halo medium model, the density distribution of the CGM could be affected by the initial temperature. We investigated the effect of the initial temperature by setting it to $2.5 \times 10^6$ K and $1.5 \times 10^6$ K (the fiducial value is $2 \times 10^6$ K). The results are shown in Supplementary Figure 5. Both cases failed in modeling the prominently asymmetric features as observed. We did not explore wider temperature range, since when $T_{\rm CGM} > 2.5 \times 10^6$ K, the background X-rays will become too bright, and when $T_{\rm CGM} < 1.5 \times 10^6$ K, the X-ray bubbles will be too dim and uneven.

**Supplementary Note 6: Effect of the Tilted Angle of Nuclear Outflow**

In the tilted nuclear outflow model, we have tested different tilted angles $\alpha_{\rm out}$, and presented the cases

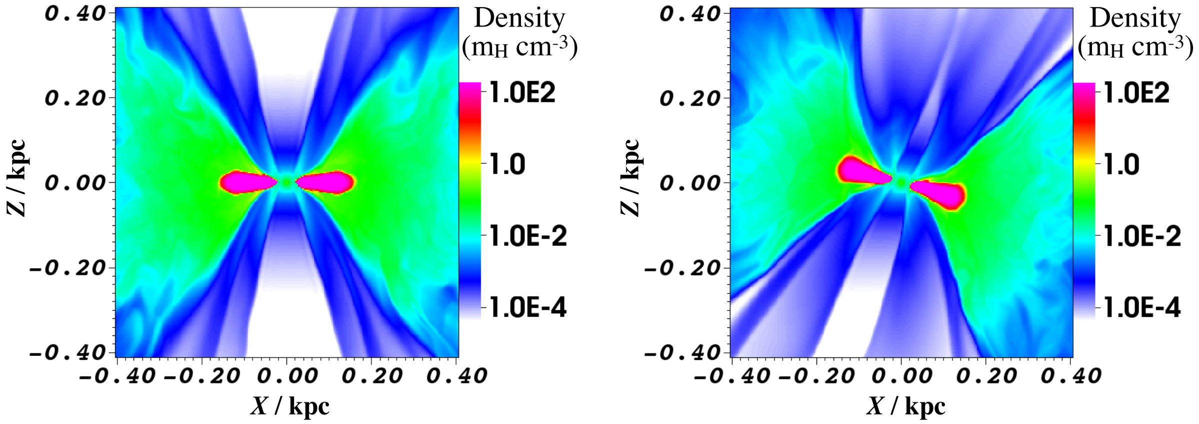


**Supplementary Figure 2. Small-scale simulations.** Left: all models except for the titled nuclear outflow model. Right: the titled nuclear outflow model ($\alpha_{\text{out}} = 17°$ case). Coordinate values are in units of kpc.

of $\alpha_{\text{out}} = 7°$ and $37°$ in Supplementary Figure 6. The simulated northern X-ray bubble exhibits a brighter edge on the right/east side due to stronger compression. Moreover, the outline of the northern bubble does not exhibit prominent asymmetry as observed even when $\alpha_{\text{out}} = 37°$, suggesting that the asymmetry of the bubble's outline is insensitive to the nuclear outflow's direction. This remains true even when considering rotational CGM (see below).

## Supplementary Note 7: Effect of the CGM Rotation

We have tested the effect of rotating CGM on the results (Supplementary Figure 7) and investigated three different rotational speed: $f = 0.3, 0.5$ and $0.7$ (see Method for the definition of $f$). Apparently, in the non-axisymmetric halo medium and tilted nuclear outflow model, the X-ray bubbles will become narrower in the east–west direction when $f$ is higher. The reason is that when rotation is faster, the density distribution in the lateral direction will be flatter (see Equation 5), resulting in a greater resistance for the bubble to expand laterally. All of these cases failed in reproducing the prominently asymmetric NeRB as observed. In addition, when rotation is incorporated, the initial CGM forms a low-density channel near the Galactic pole. Therefore, in the titled nuclear outflow model, the top of the bubble gradually moves towards to the Galactic pole due to lower resistance, forming a ")"–shaped cavity in the northern halo which is contrary to the "("–shaped northern PRL (see the panels of $f = 0.5$ in Supplementary Figure 7).

For the CGM wind model, the situation is more complicated, since the rotation of the halo medium is coupled with the parameters of the CGM wind, such as the angular momentum of the CGM wind, the CGM wind velocity $v_{\text{CGM}}$, etc. Here we only adjust one parameter of $v_{\text{CGM}}$, and pick out the results that match the observations as closely as possible (judging by the eyes on the projection 0.6–1.0 keV surface brightness) and show them in Supplementary Figure 8. According to these preliminary tests, the results of modeling eRBs are not significantly improved.

## Supplementary Note 8: High-Velocity Clouds

In our fiducial run (at $t = 360 + 19$ Myr), we find that high-velocity clouds (HVC) form from the cooling of the incoming CGM wind. For the convenience of comparison with observations[5], the clouds are projected onto the sky in the Galactic Coordinate System with the anti-GC in the center (Supplementary Figure 9). For the local standard of rest (LSR) velocity, we assume the standard orbital velocity of

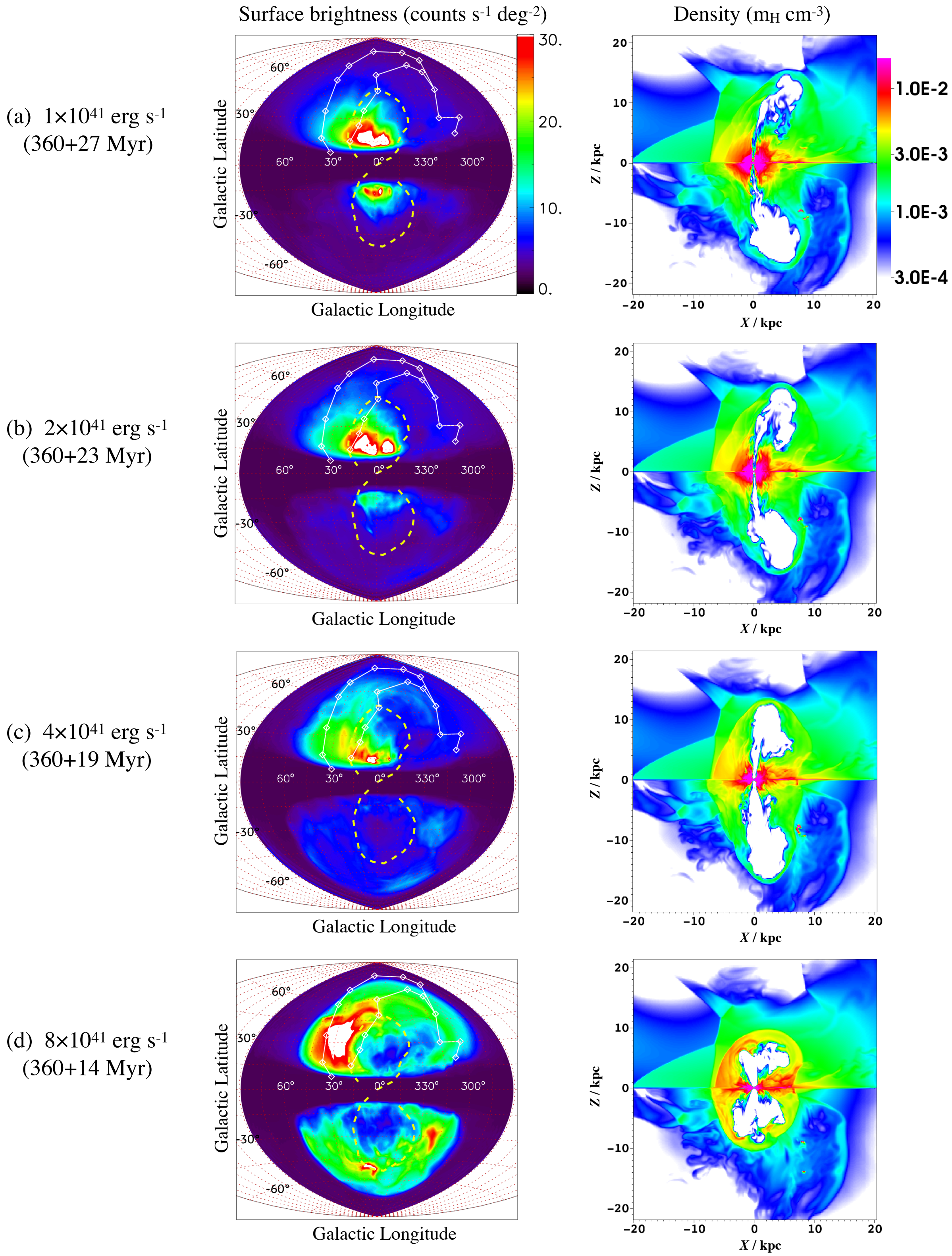


**Supplementary Figure 3. Simulation tests for the different values of $L_k$.** The left panels show the maps of X-ray surface brightness in 0.6–1.0 keV range (see the legend in the top panel). The right panels present the corresponding density profile (see the legend in the top panel).

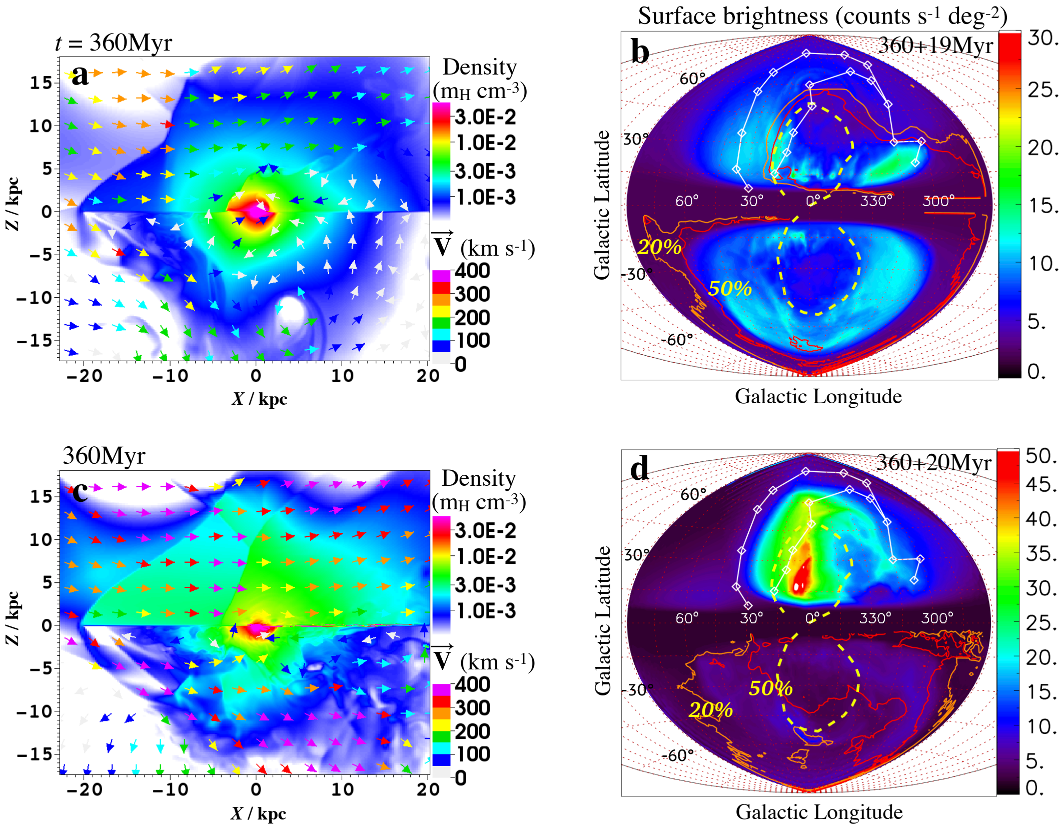


**Supplementary Figure 4. Tests for weak and strong CGM wind.** For weak CGM wind (upper panels), we set $v_{\rm CGM} = 100$ km s$^{-1}$. For strong CGM wind (lower panels), $v_{\rm CGM} = 300$ km s$^{-1}$. The orange and red solid lines represent contours of $\chi_{\rm init}$ of 20% and 50%, respectively.

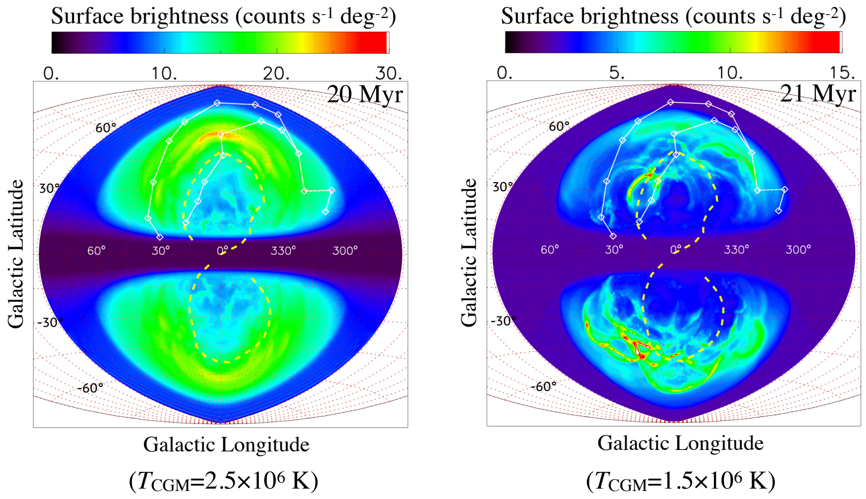


**Supplementary Figure 5. Results of different initial CGM temperatures in the non-axisymmetric halo medium model.** Note the colorbar in right panel is different from the left.

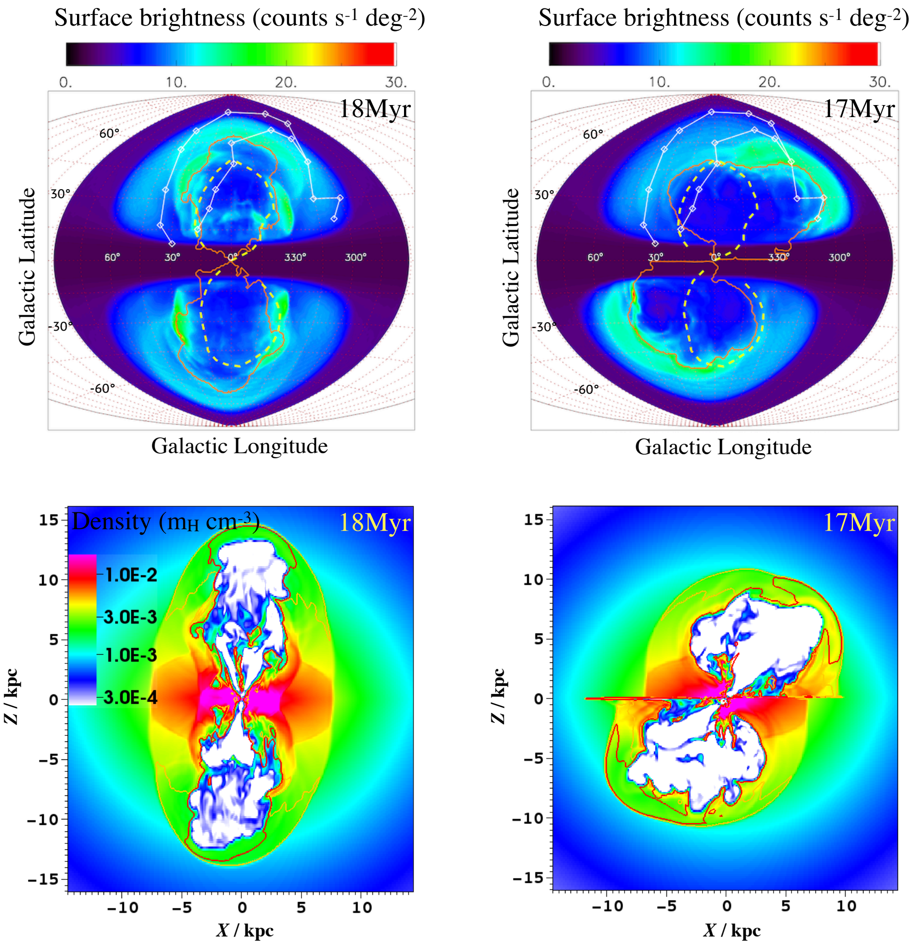


**Supplementary Figure 6.** **Results of different tilted angles.** The left and right panels show the results of $\alpha_{\rm out} = 7^\circ$ and $37^\circ$, respectively. The upper panels present the 0.6–1.0 keV surface brightness (orange lines – cavities). The lower panels present the density distribution on $Y = 0$ (orange lines – $3 \times 10^6$ K, red lines – $4 \times 10^6$ K).

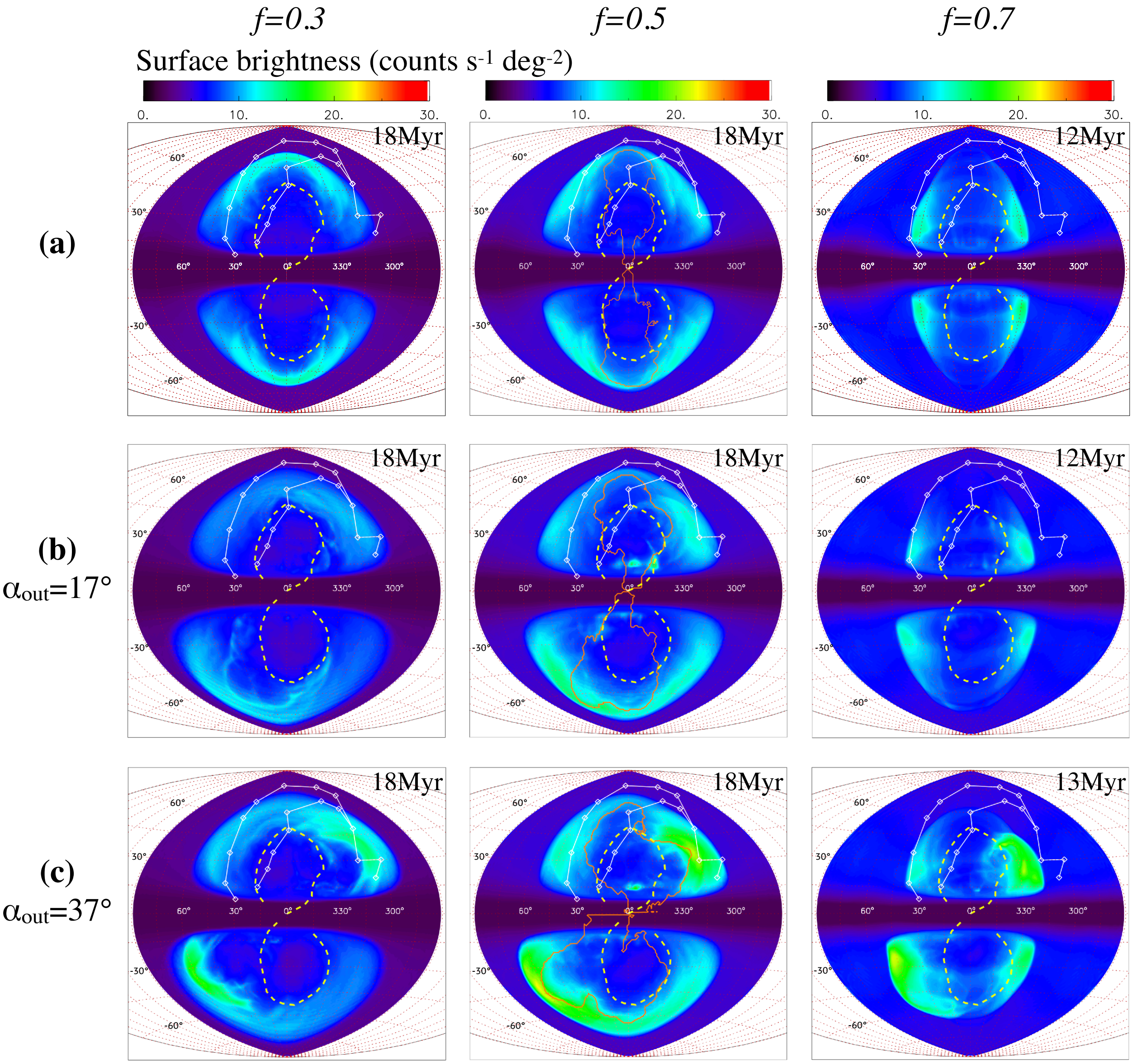


**Supplementary Figure 7. Effect of rotating CGM.** Row (a) show the non-axisymmetric halo medium models. Row (b) and row (c) show the tilted nuclear outflow models. The rotation of CGM mainly affects the width of the bubbles in the lateral direction. The orange lines in the panels of $f = 0.5$ mark the bubble cavities. All these cases failed in reproducing the prominently asymmetric NeRB. Note that for the cases with $f = 0.7$, the caps of bubbles have already crossed the boundaries of simulation box (at $t = 12$ or 13 Myr) and therefore the brightness at $|b| > 60°$ is underestimated.

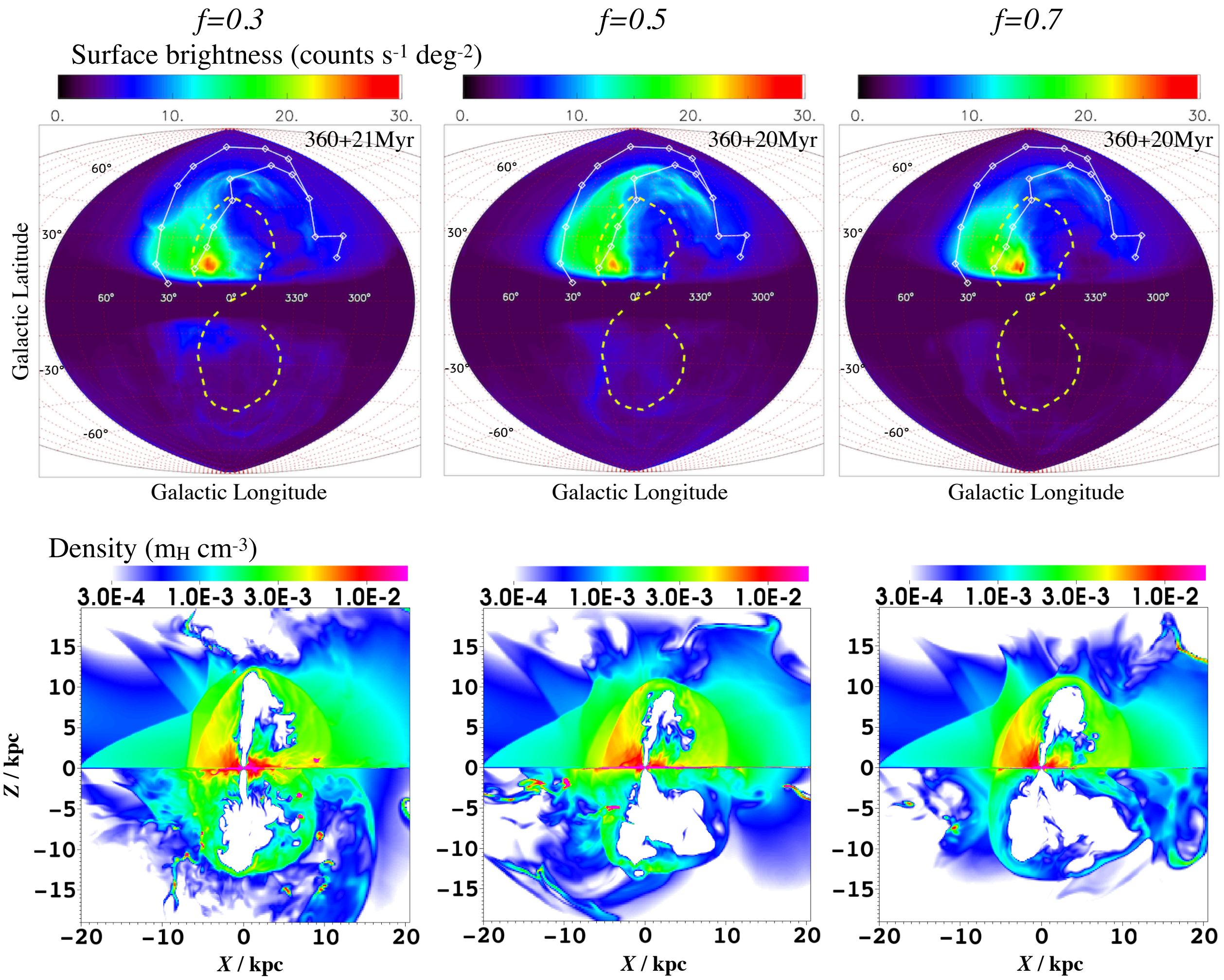


**Supplementary Figure 8.** **Effect of rotating CGM in the CGM wind model.** The upper panels present the 0.6–1.0 keV surface brightness, and the lower panels present the density distribution on $Y = 0$. $v_{\rm CGM}$ adopted here is 140 km $s^{-1}$ ($f = 0.3$), 120 km $s^{-1}$ ($f = 0.5$) and 120 km $s^{-1}$ ($f = 0.7$), respectively.

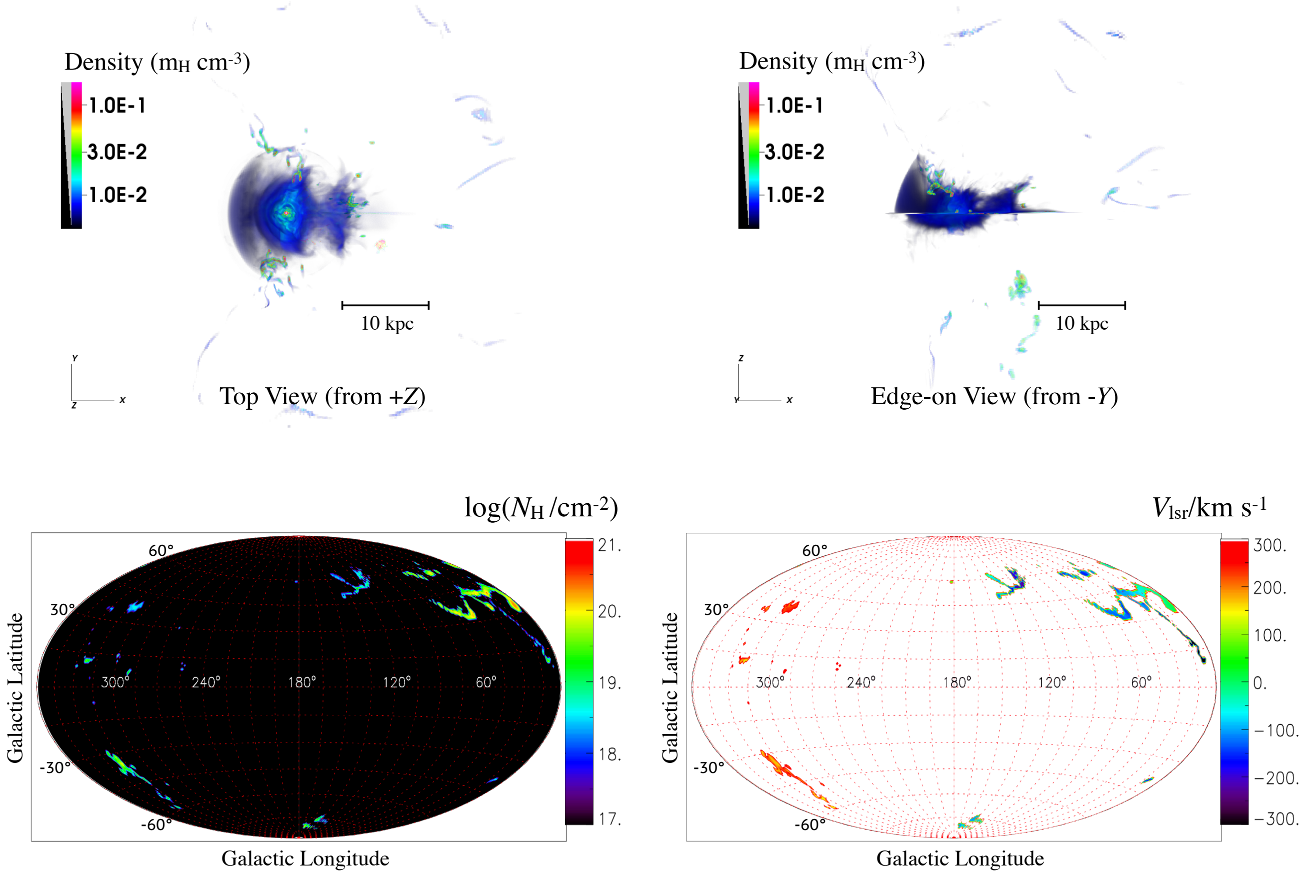


**Supplementary Figure 9. High-velocity clouds.** Upper panels show 3D views of gas density (only showing $\rho > 4 \times 10^{-3}$ $m_H$ cm$^{-3}$) at $t$ =360+19 Myr for the fiducial model. The minor tick marks on the axes are at interval of $5 \times 10^{21}$ cm (1.6 kpc). Solar system is at $(X, Y, Z) = (0, -8.2\ \text{kpc}, 0)$. Lower panels show the projected column density and LSR velocity of clouds.

$V_{sun} = 250$ km s$^{-1}$[6]. The clouds are concentrated in the quadrant of $180° > l > 0°$, $b > 0°$ (infalling region of the CGM wind). The simulated cloud complex in latitude of $0 - 75°$ roughly resembles the observed Complex C at the similar sky area. Excepting the lower part of $b < 30°$, this simulated complex exhibits a LSR velocity of 0–150 km s$^{-1}$, and a column density of $10^{19-20}$ cm$^{-2}$, which are also similar to those of the Complex C[5].

However, we should caution that parameters of the HVC given by our model are preliminary, since the uncertainties of current observations prevent us from further refining the simulation parameters. The injection area and the injection direction of the CGM wind are somewhat arbitrary. These will affect the final result on the formation of the clouds. The results on the HVC presented here is only to illustrate that the CGM wind scenario is not contradictory to the HVC, and furthermore, it may potentially explain the formation of HVC.

**Supplementary Note 9: Effect of Numerical Resolution**

We tested the effect of numerical resolutions by performing a lower resolution run (mesh size = 2×fiducial) and a higher resolution run (mesh size = 0.6×fiducial). Comparing with the results in different resolutions (Supplementary Figure 10), we find that the values of the cavity volume are 570, 480 and 460 kpc$^3$ in the low-, fiducial- and high-resolution cases, respectively, indicating that the results rapidly

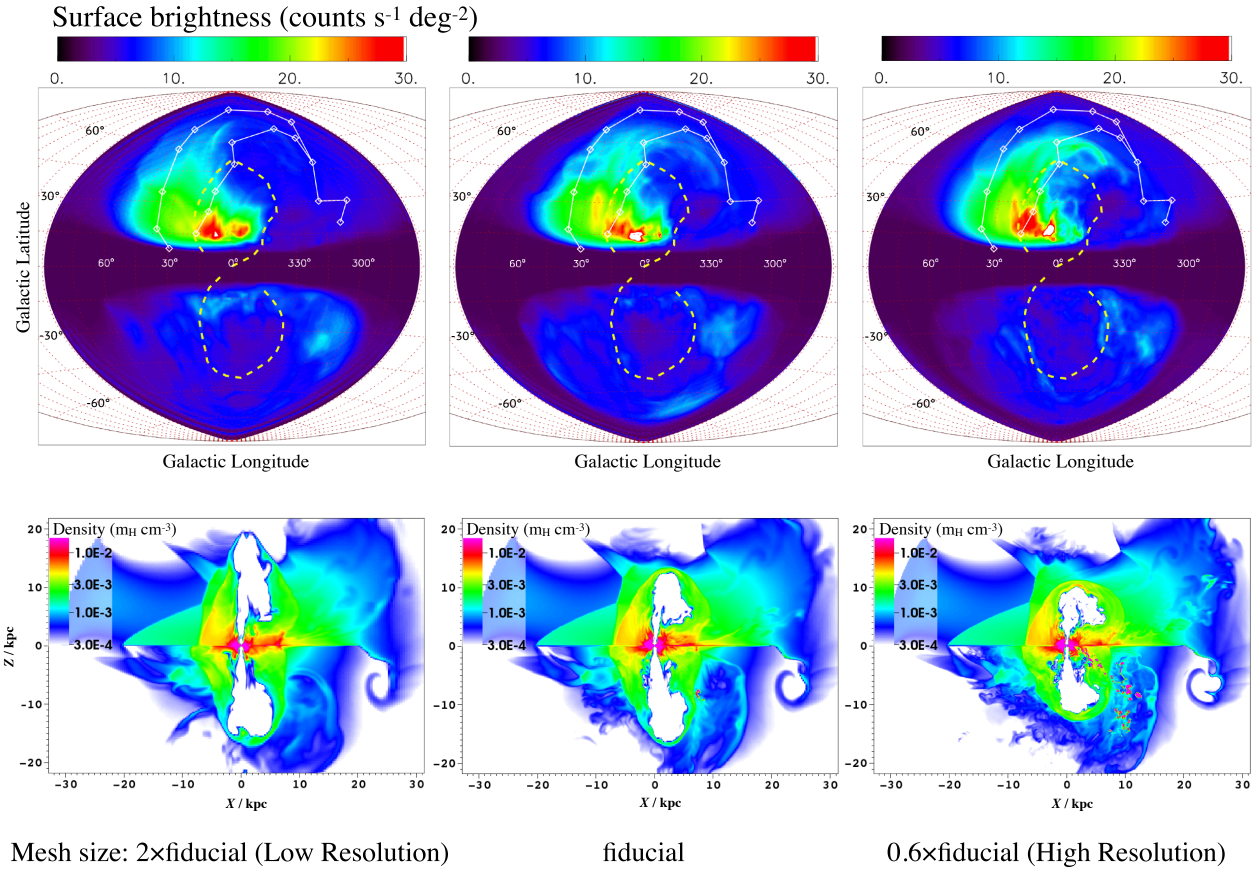


**Supplementary Figure 10. Effect of Numerical Resolution.** The upper panels present the 0.6–1.0 keV surface brightness in different resolutions, and the lower panels present the density distribution on $Y = 0$ ($t = 360 + 19$ Myr).

converge on the resolution. The effect on the bubble morphology with different resolutions are mainly due to the opening angles of the nuclear outflow injected into the halo, which are affected by the mesh size. When adopting smaller meshes (higher resolution), it is easier to capture the development of the Kelvin-Helmholtz (KH) instabilities. As the wavelength of the KH instability develops close to the size of the nozzle, the wobbling of the nozzle widens it and the opening angle becomes larger. In turn, when adopting larger meshes (lower resolution), the KH instabilities will be difficult to develop. The numerical resolution plays a viscous–like role (numerical viscosity), and KH instabilities are more easily suppressed in the low resolution case. It changes bubble's morphology by affecting the opening angle of the outflow. However, the most important parameter here is the kinetic power of the outflow (almost unchanged in all the three resolutions), while the opening angle of the nuclear outflow is a secondary effect. The effect of numerical resolution does not change the main conclusions of this work.

## Supplementary References


**1.** Henley, D. B. & Shelton, R. L. An xmm-newton survey of the soft x-ray background. iii. the galactic halo x-ray emission. *The Astrophysical Journal* **773**, 92 (2013).

**2.** Kataoka, J. *et al.* Suzaku observations of the diffuse x-ray emission across the fermi bubbles'edges. *The Astrophysical Journal* **779**, 57 (2013).

**3.** Nakahira, S. *et al.* Maxi/ssc all-sky maps from 0.7 kev to 4 kev. *Publications of the Astronomical Society of Japan* **72**, 17 (2020).

**4.** Sarkar, K. C., Nath, B. B. & Sharma, P. Multiwavelength features of fermi bubbles as signatures of a galactic wind. *Monthly Notices of the Royal Astronomical Society* **453**, 3827–3838 (2015).

**5.** Westmeier, T. A new all-sky map of galactic high-velocity clouds from the 21-cm hi4pi survey. *Monthly Notices of the Royal Astronomical Society* **474**, 289–299 (2018).

**6.** Reid, M. *et al.* Trigonometric parallaxes of massive star-forming regions. vi. galactic structure, fundamental parameters, and noncircular motions. *The Astrophysical Journal* **700**, 137 (2009).
